# Supplementary material for: Double face of cytochrome c in cancers by Raman imaging
Source: Sci Rep. 2022 Feb 8;12:2120. doi: 10.1038/s41598-022-04803-0 (PMC8826388; doi:10.1038/s41598-022-04803-0)
Supplement: Supplementary file 1 — Supplementary Information. [file 41598_2022_4803_MOESM1_ESM.docx]

**SUPPLEMENTARY MATERIALS**

Figure S1. shows the microscopy image, the Raman image, the histopathological image, the comparison of average Raman spectra obtained by Cluster Analysis Method and the Raman spectra characteristic for pure chemical components: oleic acid, β-carotene, palmitic acid, mammaglobin-A, collagen, cytochrome c, cardiolipin for normal human breast duct.


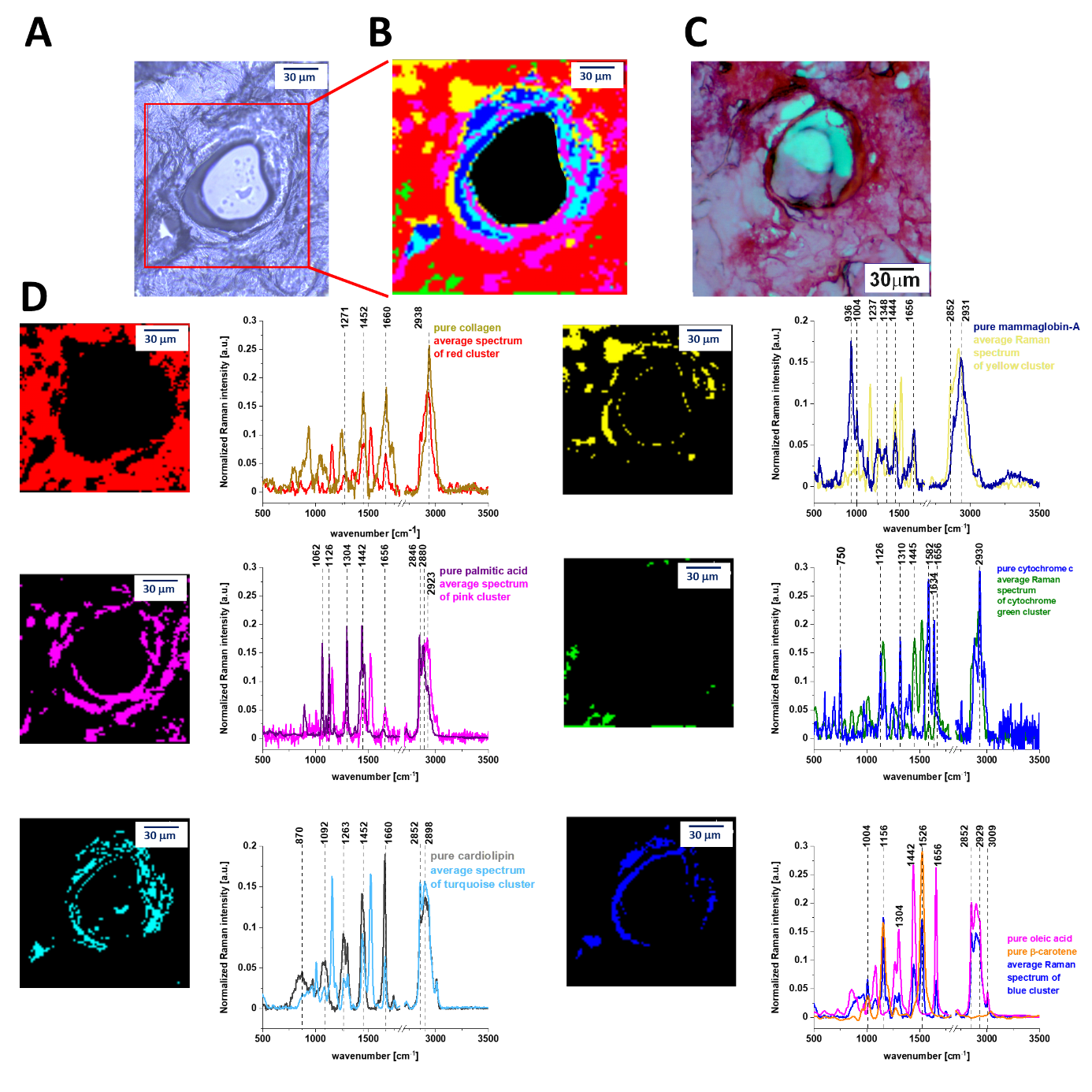


**Figure S1.** (A) Microscopy image of human normal breast duct, (B) the Raman image of human normal breast duct obtained by Cluster Analysis, (C) the histopathological image of human normal breast duct and (D) the comparison of average Raman spectra (normalized by norm) obtained by Cluster Analysis Method and the Raman spectra characteristic for pure chemical components: oleic acid, β-carotene, palmitic acid, mammaglobin-A, collagen, cytochrome c, cardiolipin.

Figure S2. the microscopy image, the Raman image, the histopathological image, the comparison of average Raman spectra obtained by Cluster Analysis Method and the Ramana spectra characteristic for pure chemical components: oleic acid, β-carotene, palmitic acid, mammaglobin-A, collagen, cytochrome c, cardiolipin for cancerous human breast duct.


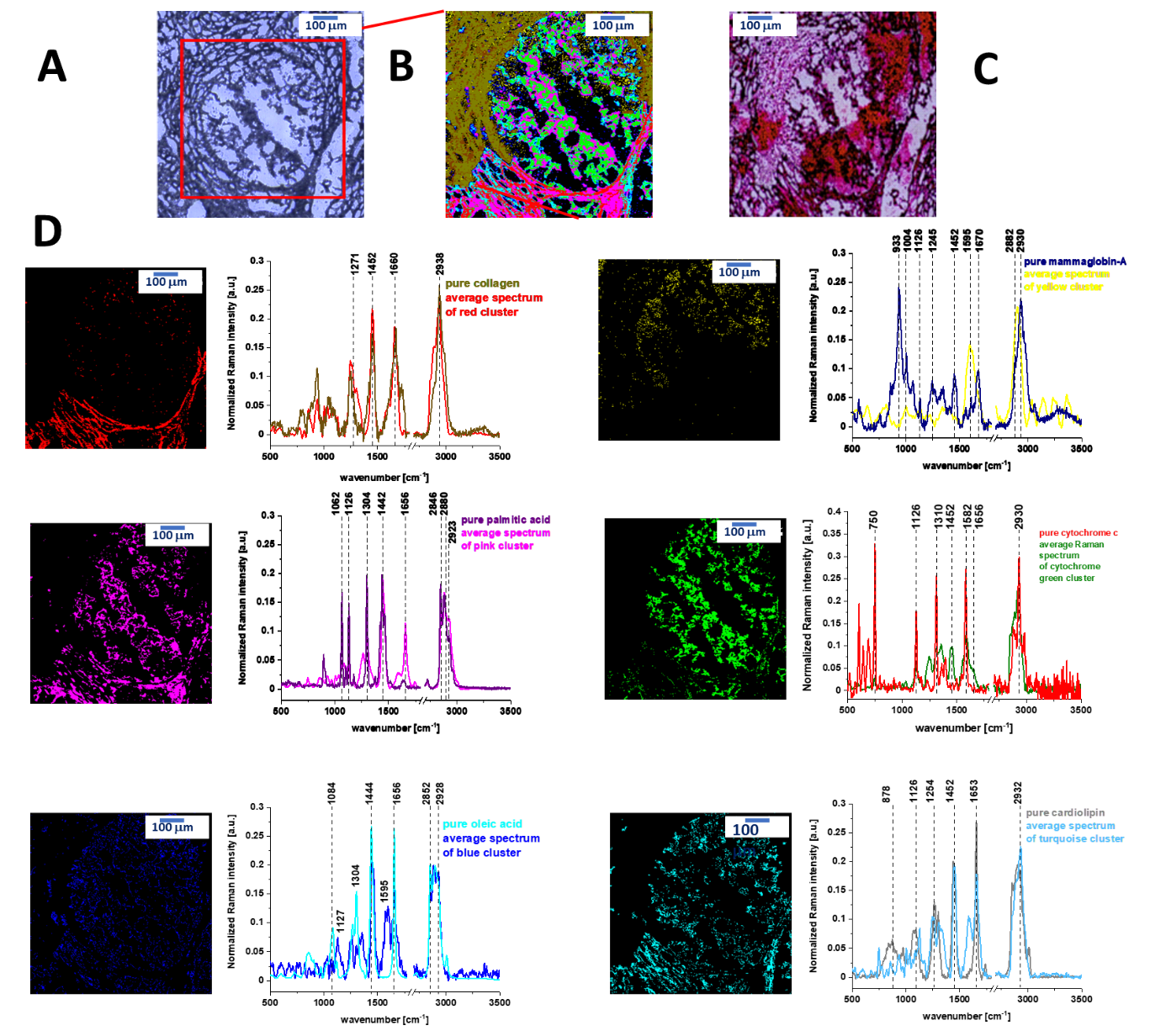


**Figure S2.** (A) Microscopy image of human cancerous breast duct, (B) the Raman image of human cancerous breast duct obtained by Cluster Analysis, (C) the histopathological image of human cancerous breast duct and (D) the comparison of average Raman spectra (normalized by norm) obtained by Cluster Analysis Method and the Raman spectra characteristic for pure chemical components: oleic acid, β-carotene, palmitic acid, mammaglobin-A, collagen, cytochrome c, cardiolipin.

Figure S3. shows the results of Basis analysis performed for normal human breast duct based on the Raman spectra of pure: oleic acid, β-carotene, palmitic acid, mammaglobin-A, collagen, cytochrome c, cardiolipin.

**
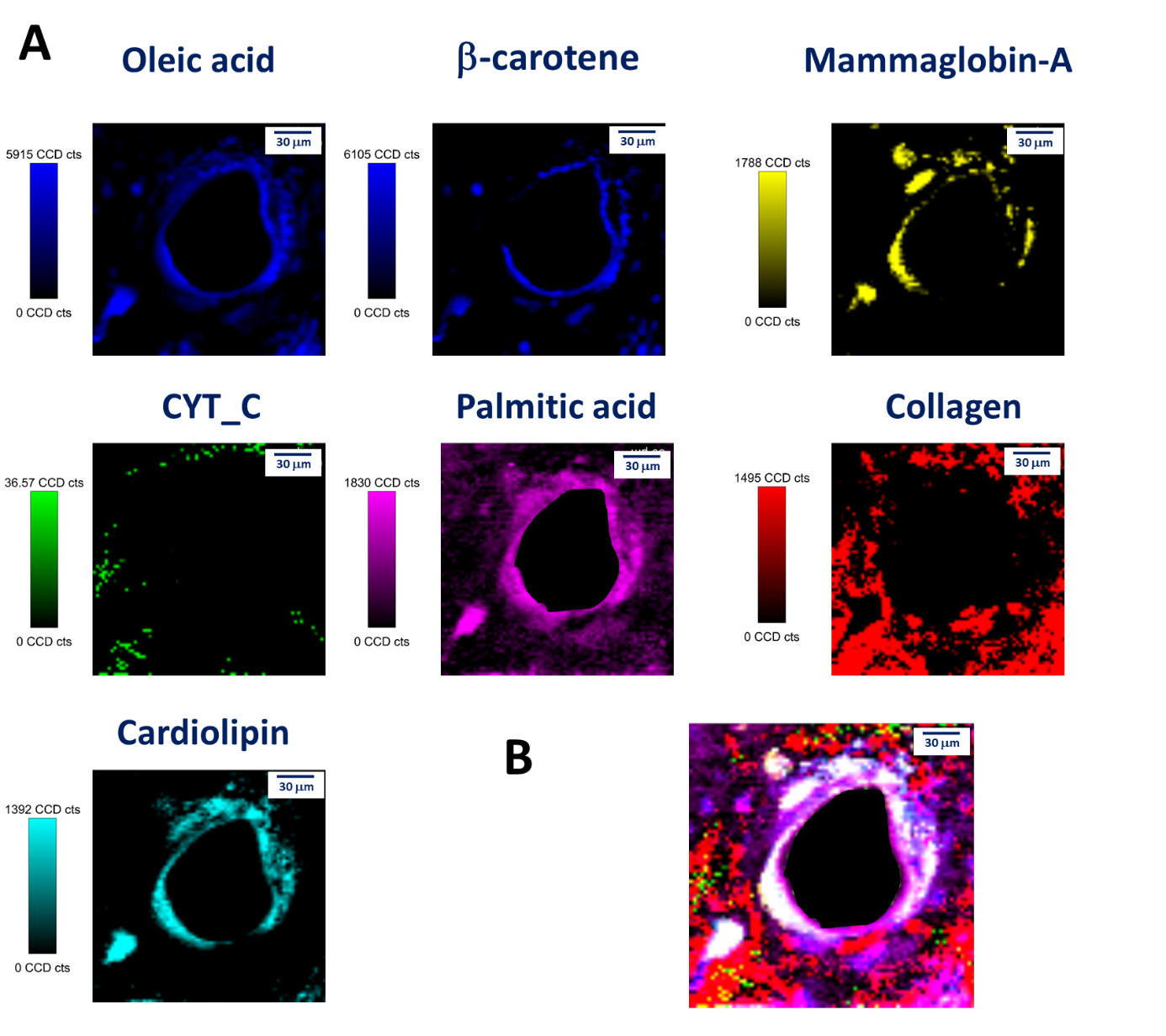
Figure S3.** (A) Basis analysis performed for normal human breast duct based on the Raman spectra of pure: oleic acid, β-carotene, palmitic acid, mammaglobin-A, collagen, cytochrome c, cardiolipin; the images obtained for single chemical components and (B) the combination of images obtained for single chemical components shown on panel (A).

Figure S4. shows the results of Basis analysis performed for cancerous human breast duct based on the Raman spectra of pure: oleic acid, palmitic acid, mammaglobin-A, collagen, cytochrome C, cardiolipin.


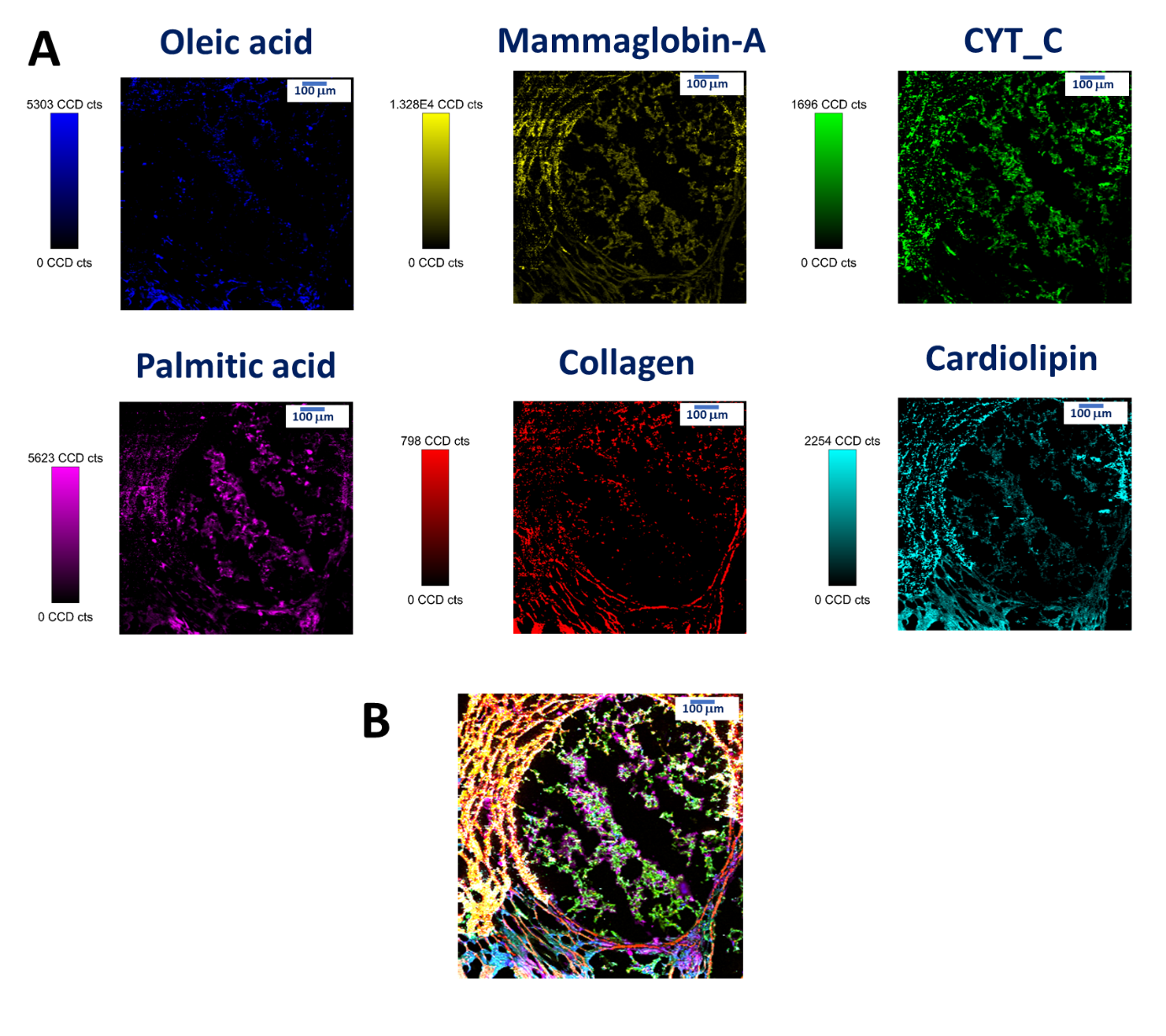


**Figure S4.** (A) Basis analysis performed for cancerous human breast duct based on the Raman spectra of pure: oleic acid, palmitic acid, mammaglobin-A, collagen, cytochrome c, cardiolipin; the images obtained for single chemical components and (B) the combination of images obtained for single chemical components shown on panel (A).

Figure S5 shows the microscopy image (A), the Raman image of a single cell MCF7 of breast cancer obtained by Cluster Analysis (B) and corresponding average Raman spectra of different organelles of the cell: nucleus (red), cell membrane (light grey), lipid structures (blue and orange), cytoplasm (green) and mitochondria (magenta).

**
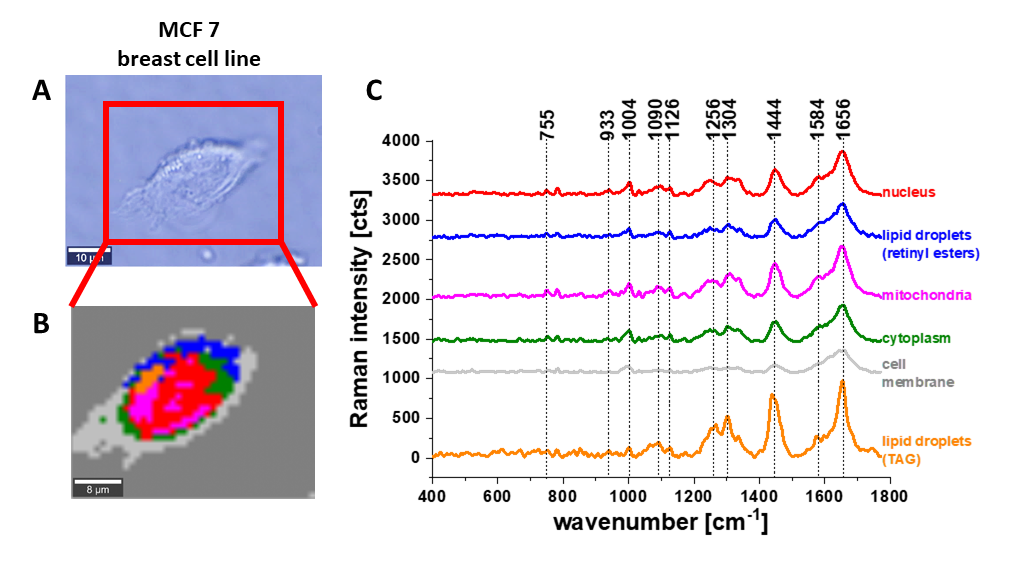
**

**Figure S5.** (A) The microscopy image, (B) the Raman image of a single human cancerous epithelial cell MCF7 obtained by Cluster Analysis and (C) the corresponding average Raman spectra of different organelles of the cell: mitochondria (magenta), lipid droplets (TAG) (orange), lipid droplets (retinyl esters) (blue), cytoplasm (green), nucleus (red), cell membrane (light grey).

Table 1 presents the Pearson correlation coefficients obtained for comparison of the average Raman spectra typical for normal and cancerous human duct and the Raman spectra characteristic for pure components such as: oleic acid, β-carotene, palmitic acid, mammaglobin-A, collagen, cytochrome c, cardiolipin.

|  | **NORMAL HUMAN DUCT** | **The average Raman spectrum** | **CANCEROUS HUMAN DUCT** | **The average Raman spectrum** |
| --- | --- | --- | --- | --- |
|  | **Pearson correlation coefficient** | **p-value** | **Pearson correlation coefficient** | **p-value** |
| Cytochrome C reduced form | 0.99688 | <0.05 | 1.00000 | <0.05 |
| Cytochrome C oxidized form | 0.99965 | <0.05 | - | - |
| Collagen | 0.99996 | <0.05 | 0.99999 | <0.05 |
| Mammaglobin-A | 0.99917 | <0.05 | 0.99928 | <0.05 |
| Palmitic acid | 0.99999 | <0.05 | 1.00000 | <0.05 |
| Oleic acid,  β-carotene | 1.00000 | <0.05 | - | - |
| Oleic acid | - | - | 0.99997 | <0.05 |
| Cardiolipin | 0.99999 | <0.05 | 0.99999 | <0.05 |

**Table 1**. Pearson correlation coefficients obtained for comparison of the average Raman spectra typical for normal and cancerous human breast duct and the Raman spectra characteristic for pure components such as: oleic acid, β-carotene, palmitic acid, mammaglobin-A, collagen, cytochrome c, cardiolipin.
